# Analyses of single nucleotide polymorphisms identified by ddRAD-seq reveal genetic structure of tea germplasm and Japanese landraces for tea breeding

**DOI:** 10.1371/journal.pone.0220981

**Published:** 2019-08-08

**Authors:** Hiroto Yamashita, Hideyuki Katai, Lina Kawaguchi, Atsushi J. Nagano, Yoriyuki Nakamura, Akio Morita, Takashi Ikka

**Affiliations:** 1 Faculty of Agriculture, Shizuoka University, Ohya, Shizuoka, Japan; 2 United Graduate School of Agricultural Science, Gifu University, Yanagito, Gifu, Japan; 3 Shizuoka Prefectural Research Institute of Agriculture and Forestry, Tea Research Center, Kurasawa, Kikugawa, Shizuoka, Japan; 4 Faculty of Agriculture, Ryukoku University, Yokotani, Seta Oe-cho, Otsu, Shiga, Japan; 5 School of Food and Nutritional Sciences, University of Shizuoka, Yada, Shizuoka, Japan; National Cheng Kung University, TAIWAN

## Abstract

To obtain genetic information about the germplasm of tea (*Camellia sinensis* L.) in Japan, 167 accessions including 138 var. *sinensis* (96 Japanese var. *sinensis* and 42 exotic var. *sinensis*) and 29 Assam hybrids were analyzed using single nucleotide polymorphisms (SNPs) markers identified by double-digest restriction-site-associated DNA sequencing (ddRAD-seq) analysis. Approximately 10,000 SNPs were identified by ddRAD-seq and were mapped across the whole genome. The 167 tea accessions were classified into three genetic subgroups: (1) Japanese var. *sinensis*; (2) Japanese and exotic var. *sinensis*; (3) Assam hybrids and exotic var. *sinensis*. Leaf morphology varied widely within each genetic subgroups. The 96 Japanese var. *sinensis* were classified into four genetic subgroups as follows; two subgroups of Shizuoka (the largest tea production region) landraces, Uji (most ancient tea production region) landraces, and the pedigree of ‘Yabukita’, the leading green tea cultivar in Japan. These results indicated that the SNP markers obtained from ddRAD-seq are a useful tool to investigate the geographical background and breeding history of Japanese tea. This genetic information revealed the ancestral admixture situation of the ‘Yabukita’ pedigree, and showed that the genome structure of ‘Yabukita’ is clearly different from those of other Japanese accessions.

## Introduction

Tea is one of the most popular beverages worldwide. The tea plant (*Camellia sinensis* (L.) O. Kuntze) contains many functional ingredients such as theanine and catechins [1]. Consequently, the production area and yield of tea are increasing worldwide, but especially in Asia (http://faostat.fao.org/). Tea is a woody plant in the Theaceae family of angiosperms. The origin of tea plants is considered to be southwestern China and surrounding regions [2–4]. In general, there are two varieties of tea plants; var. *sinensis* (Chinese type), and var. *assamica* (Assam type) [5]. These two varieties have different phenotypic characteristics and different origins [6,7]. For example, *C*. *sinensis* var. *sinensis* has small leaves and is able to withstand colder climates, while *C*. *sinensis* var. *assamica* has large leaves and is sensitive to cold temperatures. *C*. *sinensis* var. *sinensis* is mainly cultivated in China and Japan for green tea production and *C*. *sinensis* var. *assamica* is mainly cultivated in India and Sri Lanka for black tea production. Assam hybrids, which are crosses between var. *sinensis* and var. *assamica*, have larger leaves and are more cold-tolerant than var. *assamica*, and so these hybrids are mainly utilized for breeding and for black tea production in Japan and China [8]. The relationship between genetic differentiation and phenotypic characteristics such as morphological leaf traits in these tea varieties is still unclear.

Japan is one of the main green tea production areas. The tea cultivation system is more efficient in Japan than in other areas because of the use of clonal cultivars. In Japan, most tea accessions are *C*. *sinensis* var. *sinensis*. There are two hypotheses regarding the origin of the Japanese accessions: *C*. *sinensis* var. *sinensis* was introduced into Japan from China about 800 to 1,200 years ago by Buddhist priests; or, it is indigenous to Japan [3]. The results of recent studies based on DNA marker analyses support the first hypothesis [9,10].

Tea cultivation probably started in the Uji area in Kyoto in about the 13^th^ century, and then expanded throughout Japan. In Shizuoka prefecture, the main tea production area in Japan, tea seeds are said to have been introduced from China by Buddhist priests in the 13^th^ century. Therefore, most Japanese tea accessions have originated from Uji and Shizuoka landraces.

In the early 20^th^ century, ‘Yabukita’, which is now the leading tea cultivar in Japan, was selected by a tea breeder, Hikosaburo Sugiyama, from seedlings obtained by natural crossing in Shizuoka. Currently, ‘Yabukita’ is grown in about 75% of tea fields in Japan [3], and is frequently used as a parent material for breeding new cultivars. Therefore, many major Japanese tea cultivars are derived from ‘Yabukita’ [3]. Consequently, the genetic diversity of breeding populations is expected to be narrow.

It takes more than 20 years from crossing to obtain a new cultivar from several thousand pedigrees, because it takes a long time to evaluate growth and quality characteristics. Thus, tea breeding is a slow process. To obtain a new cultivar with the desired traits in a shorter period, crossing and selection based on genetic information (i.e., molecular breeding methods) should be adopted in tea breeding programs. To develop a modern tea breeding system, it is necessary to understand the genetic background and ancestral composition of parental clones.

Many genetic studies have been carried out on tea accessions using methods based on simple sequence repeat (SSR), random amplified polymorphic DNA (RAPD), and amplified fragment length polymorphism (AFLP) markers [6,8–12]. However, the genetic information gained from those studies has relatively low resolution because of the small number of polymorphisms. A recent study explored the relationships between landraces and cultivars from Kyoto and those in other regions of Japan based on SSR markers and restriction site-associated DNA sequencing (RAD-seq) analyses [13]. However, the genetic information for these Japanese landraces and cultivars is still insufficient.

High-throughput next-generation sequencing (NGS) technologies have proven to be useful for the large-scale identification of genome-wide single nucleotide polymorphisms (SNPs) [14], and RAD-seq methods are also useful for identifying many SNPs [15]. Among several RAD-seq methods, double-digest RAD-seq (ddRAD-seq) is one of the most inexpensive, and is suitable for analyzing large numbers of accessions [16]. The ddRAD-seq method has been used as a genotyping tool for a wide range of crops including *Citrus* [17], tomato [18], sweet potato [19], and onion [20]. Now that the whole genome of Chinese tea has been published [21], it is possible to use SNP information for various genetic analyses.

Since the 19^th^ century, to obtain superior black tea cultivars, tea seeds of *C*. *sinensis* var. *assamica* have been collected from India and other countries and stored at Japanese tea experimental stations. As mentioned above, the varieties and accessions of tea in Japan have diverse historical and geographical backgrounds. In this study, we characterized the genetic structure of 167 tea accessions with various genetic backgrounds based on high-density SNPs identified from a ddRAD-seq analysis. We also explored the relationship between genetic differentiation and leaf morphological traits. Finally, the genetic structure of 96 Japanese tea accessions was characterized. The genetic structure of Japanese accessions, including the leading cultivar in Japan, ‘Yabukita’, reflects their local distribution and breeding history.

## Materials and methods

### Plant materials

Tea accessions were obtained from the Tea Research Center, Shizuoka Prefectural Research Institute of Agriculture and Forestry, Kikugawa city, Shizuoka, and the Botanical Research Gardens of the ICHIMURA Foundation for New Technology, Atami city, Shizuoka. The 167 accessions comprised three subspecies: 96 Japanese var. *sinensis*, 42 exotic var. *sinensis* (originating from China and Taiwan); and 29 Assam hybrids (introduced from India and Nepal to Japan). The 96 Japanese accessions comprised 30 improved cultivars and 66 landraces (29 from Shizuoka, 19 from Uji, seven from other regions, and 11 from unknown regions). To evaluate morphological leaf traits, young leaves at the same developmental stage were harvested from 149 accessions grown under the same cultivation environment at the Tea Research Center. The ‘young leaf’ developmental stage was defined as the third leaf in young shoots with four leaves in the first flush season. Detailed additional information about the tea accessions used in this study is listed in S1 Table.

### Genotyping by ddRAD-seq

Genomic DNA was extracted from young leaves of each tea accession using a DNeasy Plant Mini Kit (Qiagen) following the manufacturer’s instructions, and then ddRAD-seq was conducted as described elsewhere [22]. Genomic DNA was digested with *Bgl* II and *Eco*R I. Sequencing of 50-bp single-end reads and the index sequences of the library was conducted using one lane of Hiseq2500 (Illumina, San Diego, CA, USA). Reads were preprocessed using Trimmomatic-0.33 with the following parameters: ILLUMINACLIP TruSeq3-PE-2.fa:2:30:10, LEADING:19, TRAILING:19, SLIDINGWINDOW:30:20, AVGQUAL:20, and MINLEN:51. After preprocessing, the remaining reads were mapped to the tea reference draft genome [20] using Bowtie2, and then the SNPs were called using Stacks (ver. 1.37) [23]. These raw SNPs data were filtered against the following thresholds: SNP call rate within a locus ≥ 0.7 and minor allele frequency (MAF) ≥ 0.05. The filtered SNP data were imputed using R package missForest [24] and used for subsequent population analyses. The RAD-Seq data have been deposited in the DDBJ Sequence Read Archive (Accession number: DRA008166). Genotyping rates within an individual also showed in S1 Table.

### Population analysis

To clarify the genetic structure, we used the Bayesian clustering algorithm, hierarchical cluster analysis (HCA), and principal component analysis (PCA). The Bayesian clustering analysis was performed using STRUCTURE ver. 2.3.4 [25,26]. In this analysis, we evaluated one to eight genetic subgroups (*K*) with ten runs per *K* value. For each run, the initial burn-in period was set to 50,000 with 20,000 Markov chain Monte Carlo iterations. The values of Δ*K* [27] were calculated to infer the optimum number of subgroups. The components of each subgroups, i.e., ancestral components determined by this STRUCTURE analysis, were compared with the origin and pedigree information for the accessions. The HCA was based on Ward’s method [28] using Euclidean distance and was conducted using the R function “hclust”. The dendrogram was visualized using R package ape var. 5.2, ggtree ver. 1.14.4. and ggplot2 ver. 3.1.0. The PCA was performed using the R function “prcomp”. The PC scores were plotted using R package ggplot2 ver. 3.1.0.

### Phenotyping of leaf morphological traits

Leaf shape and area as leaf morphological traits were phenotyped by image analyses. The images of sampled leaves were acquired at 200 dpi using a scanner (CanoScan LiDE 210 JP, Canon). Phenotypes of leaf shape were analyzed based on Elliptic fourier descriptors (EFDs) in the software SHAPE [29]. The contour coordinates of the leaves were extracted by image analysis and recorded as chain-codes [30]. The leaf shape was approximated by the first 20 harmonics, which corresponded to the 77 coefficients of normalized Fourier descriptors. To summarize the information contained in the coefficients of the Fourier descriptors, we performed PCA based on a variance-covariance matrix of the coefficients. The variation in shape accounted for by each component was visualized by inverse Fourier transformation [31,32]. Leaf area was quantified using LIA32 software (https://www.agr.nagoya-u.ac.jp/~shinkan/LIA32/). A heat map was generated to estimate the phenotypic population structure of morphological leaf traits, the main four variables of leaf shapes based on EFDs (PC1–4) and area, and visualized by the “heatmap.2” function of the R package gplots ver. 3.0.1. This heat map connected the hierarchical clusters based on genotypes.

## Results

### SNP identification by ddRAD-seq analysis

To acquire high-resolution genotypic information for the 167 tea accessions, we conducted SNPs genotyping by the ddRAD-seq method. After preprocessing the data, 1,269,648 SNPs were initially identified by the Stacks pipeline. Further filtering (SNP call rate within a locus ≥ 0.7, minor allele frequency (MAF) ≥ 0.05) returned 13,715 and 12,787 SNPs in all 167 accessions and the 96 Japanese accessions, respectively. To verify the locations of the detected SNPs on linkage groups, scaffolds with these SNPs were mapped to the reference tea genetic map [6,21]. In total, 11,257 of the detected SNPs in all accessions and 10,481 SNPs in the Japanese accessions were anchored across 15 linkage groups (Table 1). Because these SNPs were mapped widely across the whole genome, they could be used to gain an overview of the genetic background of each accession.

**Table 1:** Summary of SNPs on each linkage group detected by ddRAD-seq analysis.

Linkage group	SNPs number		Genetic distance (cM)	Scaffold number
	All accessions	Japanese accessions		
LG01	2,726	2,525	401.555	645
LG02	1,250	1,159	246.687	411
LG03	1,767	1,633	402.17	663
LG04	966	892	240.872	563
LG05	863	818	267.309	500
LG06	643	610	296.06	489
LG07	535	495	220.566	409
LG08	660	628	309.937	633
LG09	216	201	189.345	331
LG10	471	432	248.538	623
LG11	284	270	255.334	397
LG12	261	238	279.757	508
LG13	265	239	235.932	490
LG14	200	189	185.759	394
LG15	150	152	180.162	364
Total LG	11,257	10,481	3,959.98	7420
Not anchoring	2,458	2,306	NA	6,631
Total	13,715	12,787	NA	14,051

### Genetic structure of worldwide tea accessions

**Fig 1:**
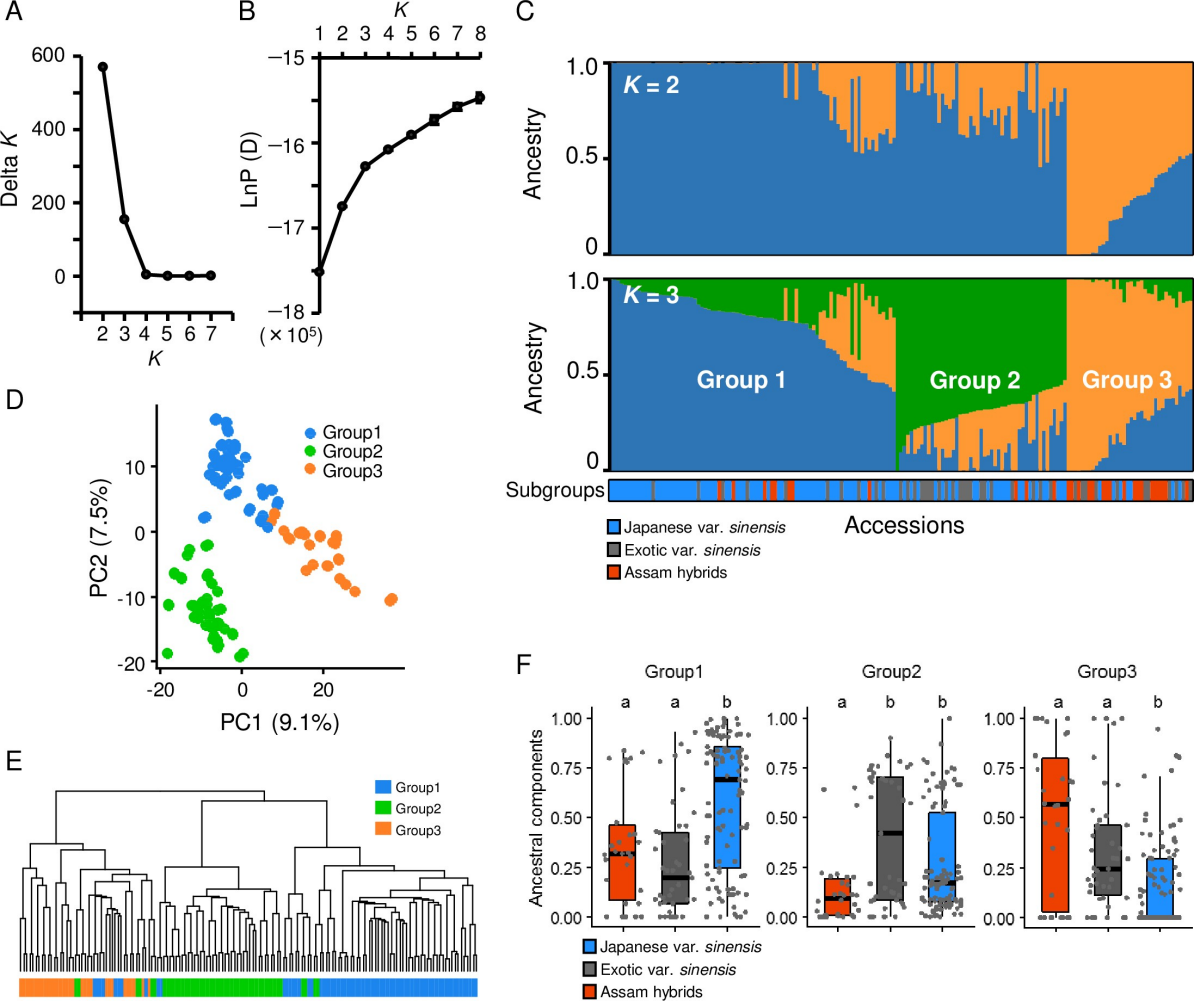
Genetic structure among worldwide 167 tea accessions. Inference of optimal *K* value by plotting of Δ*K* (A) and mean likelihood±S.D. (ten replicates) at each *K* (B). Estimated genetic structure at *K* = 2 and *K* = 3 (C). Plot of first and second principal components by PCA (D). Dendrogram of Ward’s hierarchical clustering based on Euclidean distance (E). Comparisons of distributions of three ancestral components among subgroups of Japanese var. sinensis, exotic var. sinensis, and Assam hybrids (F). Different letters above boxplots indicate significant differences (Steel-Dwass test, *P* < 0.05). Population analysis was performed with 13,715 SNPs among worldwide 167 tea accessions.

To clarify the genetic structure and the degree of relatedness among tea accessions with different genetic backgrounds, we first used the Bayesian clustering approach with high-density SNP markers. To infer the optimal number of subgroups (*K*), we calculated Δ*K* values. We obtained the highest Δ*K* values in the order of *K* = 2 and *K* = 3 (Fig 1A). At *K* = 2, the 167 worldwide tea accessions were divided into var. *sinensis* and Assam hybrids, reflecting the two main subspecies of tea plants (Fig 1C). The Δ*K* method of Evanno et al. (2005) [27] often results in *K* = 2, because there is a very low likelihood of *K* = 1 in all analyses [33]. Accordingly, there was a very low likelihood of *K* = 1 in our analyses, and the changes of likelihood values based on *K* also became moderate at *K* = 3 (Fig 1B). Therefore, we regarded *K* = 3 as the optimal classification in the genetic structure analysis of worldwide tea accessions. This classification of *K* = 3 more accurately reflected the genetic background of the tea accessions (Fig 1C) and was supported by the results of a principal components analysis (PCA) and hierarchical cluster analysis (HCA) (Fig 1D and 1E). Comparisons of the accessions with the three ancestral components at *K* = 3 revealed the main composition of each subgroups (Fig 1F): (1) Japanese var. *sinensis* (blue); (2) Japanese and exotic var. *sinensis* (green); and (3) Assam hybrids and exotic var. *sinensis* (orange).

### Genetic variation in morphological leaf traits and its relationship with genetic structure

**Fig 2:**
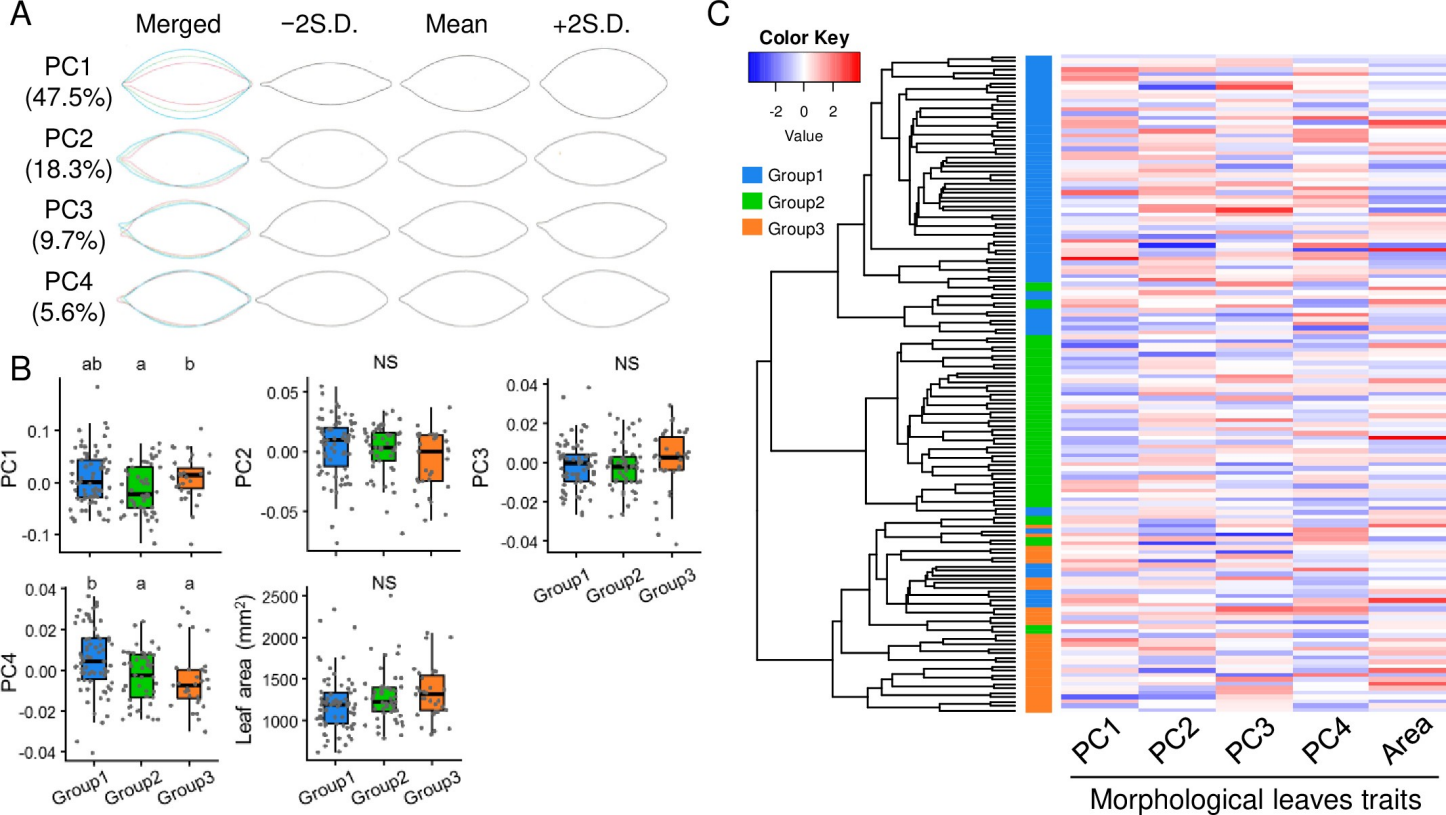
Relationship between morphological leaf traits and genetic structure in tea accessions. Variation in leaf shape accounted for by first four principal components (PCs) calculated from Elliptic fourier descriptors (EFDs) (A). Leaf shapes reconstructed from coefficients calculated by making the score for corresponding PC be equal to its mean (green) or its mean plus (blue) or minus (red) two times the standard deviation. Values in parentheses show the contribution rate of each PC. Distribution of morphological traits of tea leaves among three population groups. (B). Different letters or NS (not significant) above boxplots indicate significant differences (Steel-Dwass test, *P* < 0.05). Ward’s hierarchical clustering based on Euclidean distance between genotypes and heat map of scaled morphological traits of tea leaves (C).

To explore the relationship between genetic differentiation and morphological leaf traits in the tea accessions, we quantified morphological leaf traits based on Elliptic fourier descriptors (EFDs) and PCA. The cumulative contribution of the first four principal components (PCs) of the coefficient of EFDs accounted for more than 80% of the total variance (Fig 2A). The first four PCs represented the following visible leaf attributes (Fig 2A): PC1 represented the length to width ratio of the leaf centroid; PC2 represented the length to width ratio of the leaf tip and base; PC3 represented curvature; and PC4 represented leaf tip sharpness.

To explore the relationship between morphological leaf traits and genetic structure in tea accessions, we compared the PC values, mean values for leaf shape variables, and leaf area among the three genetic subgroups. The PC1 values were significantly higher in Group 3 than in Group 2. The PC4 values were significantly higher in Group 1 than in Groups 2 and 3 (Fig 2B and 2C). The PC2 values, PC3 values, and leaf area did not differ significantly among the genetic subgroups (Fig 2B and 2C). However, there was wide genetic variation in each morphological leaf trait within each genetic subgroups (Fig 2B and 2C).

### Genetic structure of Japanese var. *sinensis*

**Fig 3:**
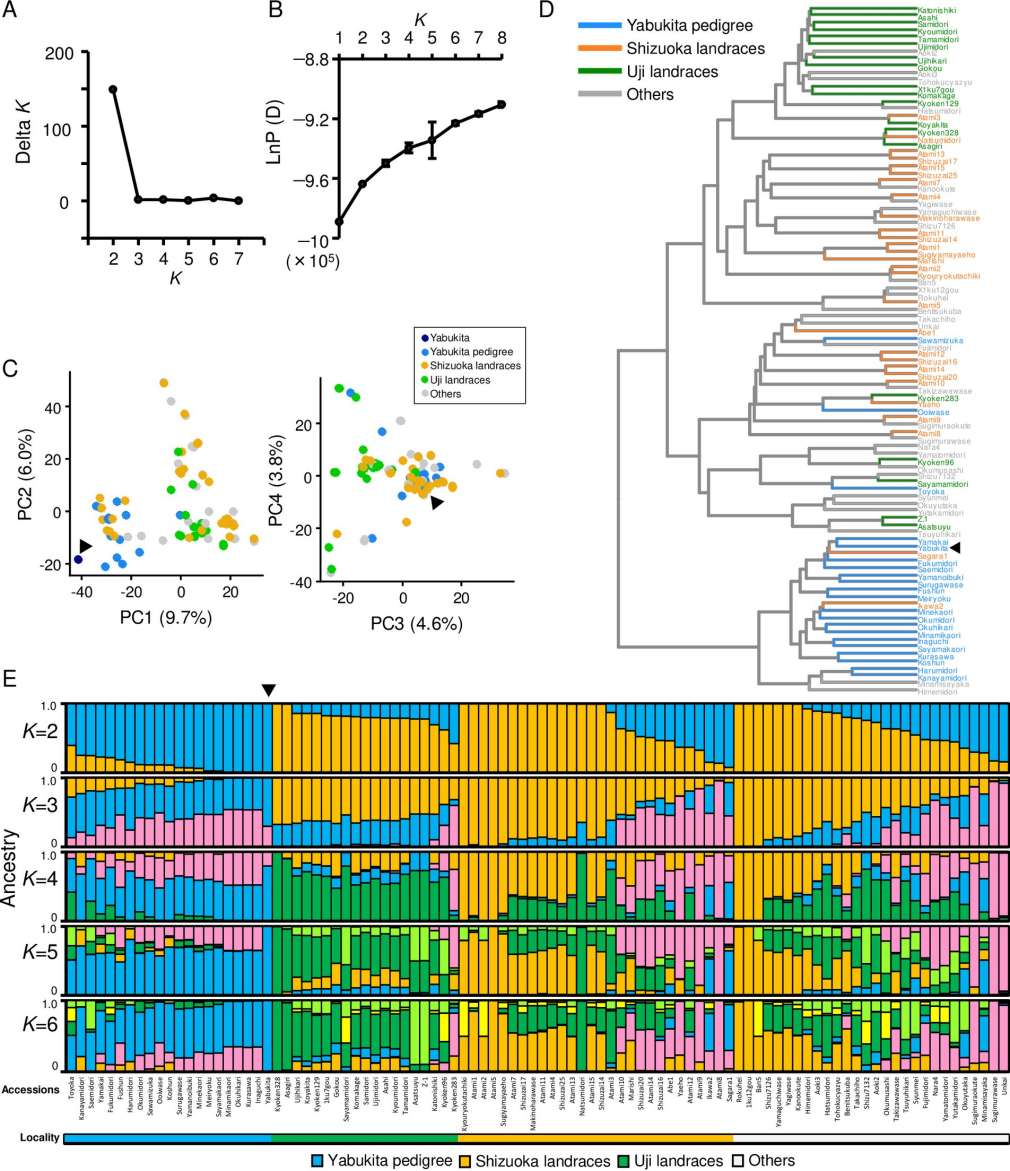
Genetic structure among 96 Japanese accessions. Inference of optimal *K* value by plotting of Δ*K* (A) and mean likelihood±S.D. (ten replicates) at each *K* (B). Plot of the first and second principal components by PCA (C). Dendrogram of Ward’s hierarchical clustering based on Euclidean distance (D). Barplots of estimated population structure from *K* = 2 to *K* = 6 (E). Locality information for each Japanese accession is shown below barplots. Population analysis was performed with 12,787 SNPs among 96 Japanese accessions. Black triangles in (C), (D) and (E) represent ‘Yabukita’.

**Fig 4:**
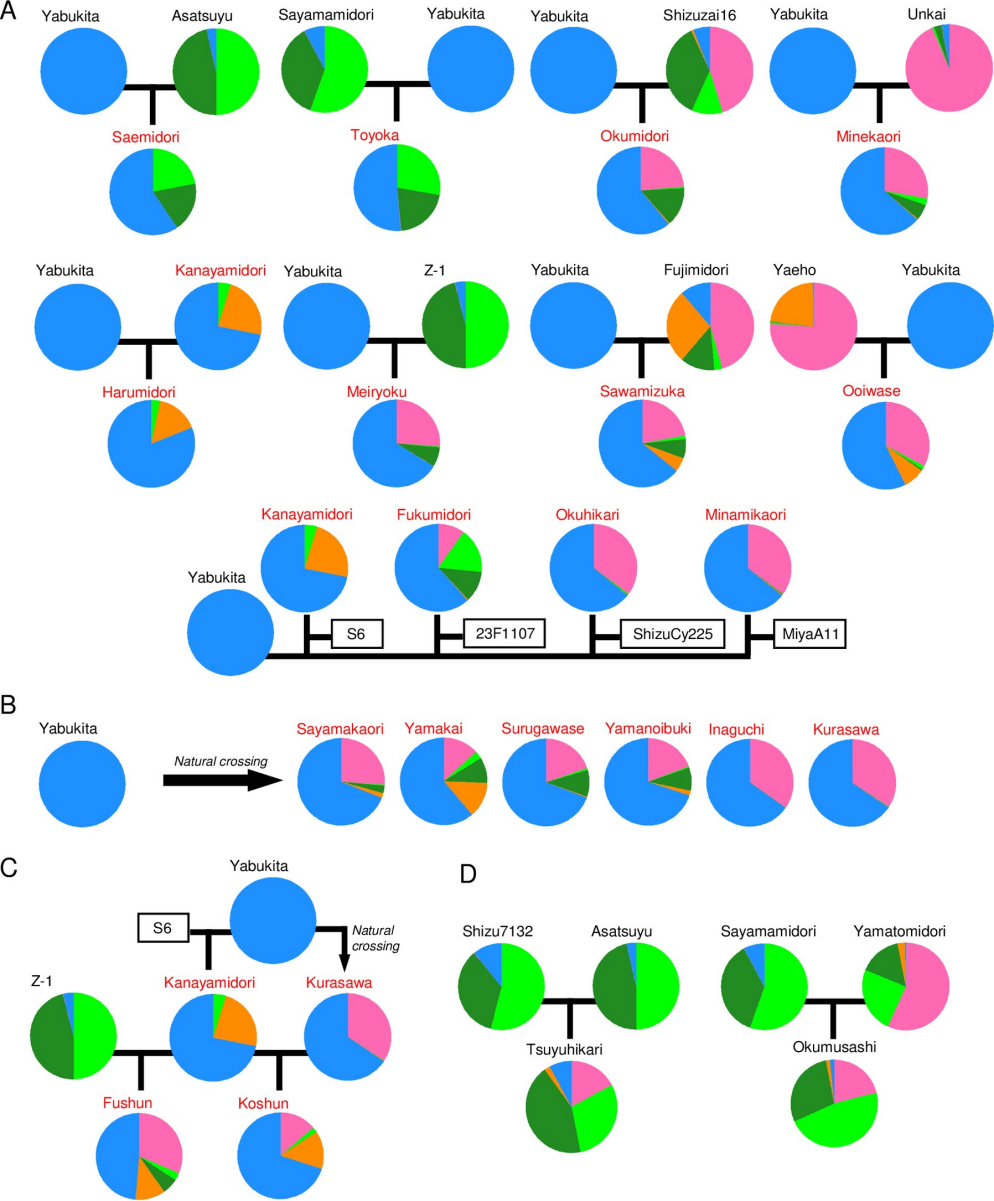
Admixture situations of improved cultivars and their parental cultivars among Japanese accessions used in this study. F1 progeny of ‘Yabukita’ from crosses (A) and natural crosses (B). F2 progeny of ‘Yabukita’ (C). These cultivars were derived from crosses between other parent materials different from ‘Yabukita’ (D). Different colors represent five ancestral components at *K* = 5 in structure analysis of Japanese var. *sinensis*. Light blue indicates the ‘Yabukita’-type ancestral component. The cultivars names of ‘Yabukita’ pedigree indicates red font.

To clarify the genetic structure and determine the degree of relatedness among Japanese tea cultivars and landraces, we conducted a Bayesian clustering analysis and multivariate analyses by PCA and HCA for the Japanese var. *sinensis*. To infer the optimal number of subgroups (*K*), we calculated Δ*K* and the likelihood values at each *K*. Although the value of Δ*K* was highest at *K* = 2 (Fig 3A), as mentioned above, these analyses have a very low likelihood of *K* = 1 (Fig 3B). However, we did not detect higher Δ*K* values for any *K* than for *K* = 2. We conducted PCA and HCA based on the SNP data for the Japanese var. *sinensis*. The results revealed the genetic differentiation among Shizuoka landraces, Uji landraces, and ‘Yabukita’ and its pedigree (Fig 3C and 3D). The barplot at *K* = 4 also reflected this genetic differentiation mainly corresponding to the two ancestral components of Shizuoka landraces (orange and pink), the ancestral component of Uji landraces (green), and the ancestral component of ‘Yabukita’ and its pedigree (blue) (Fig 3E). The barplot at *K* = 5 represents the genetic structure of improved cultivars through the breeding history of tea in Japan (Fig 4).

### Admixture situation in ‘Yabukita’, the leading tea cultivar in Japan, and its pedigree

**Fig 5:**
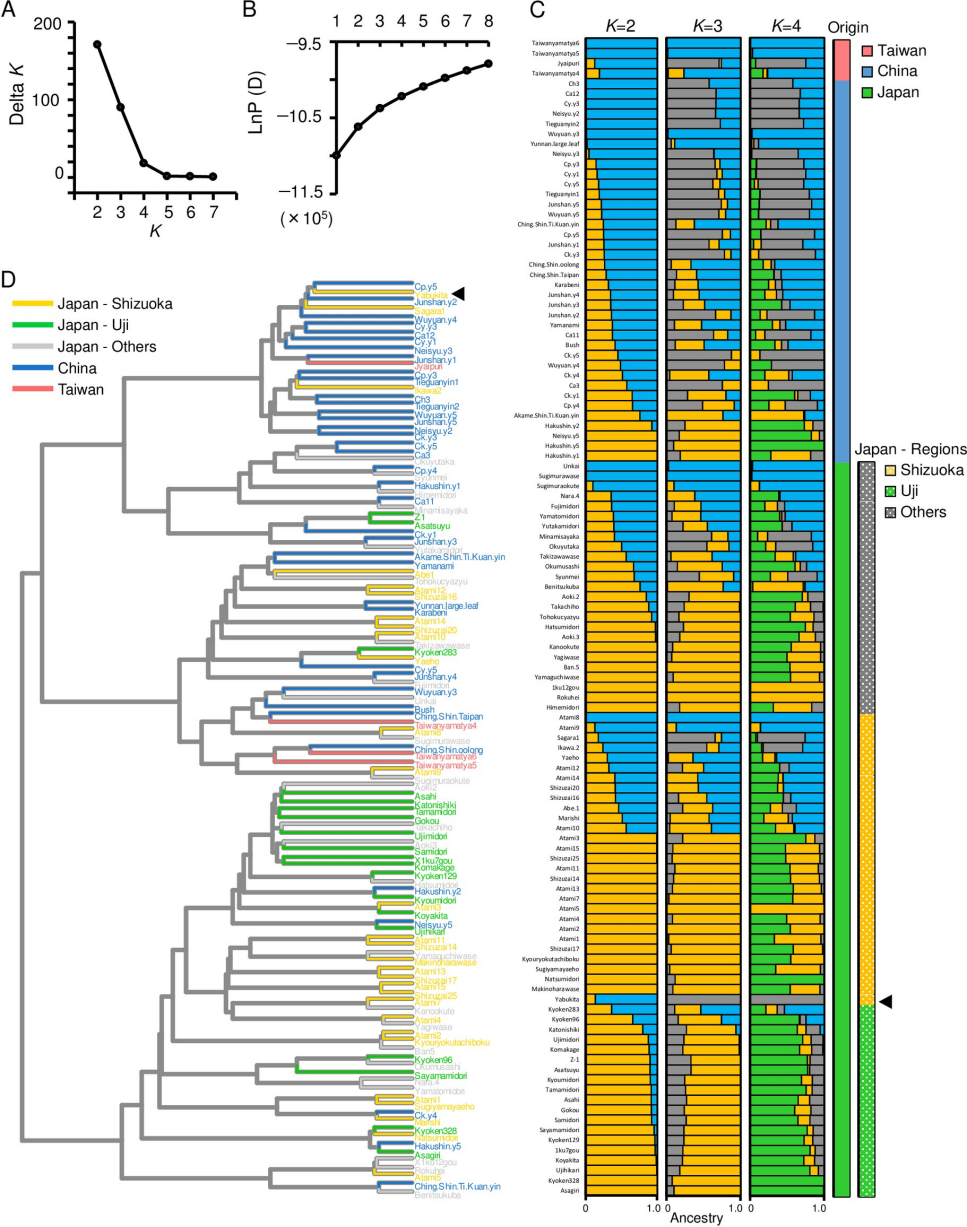
Genetic population structure among 115 var. *sinensis* accessions. Inference of optimal *K* value by plotting of Δ*K* (A) and mean likelihood±S.D. (ten replicates) at each *K* (B). Barplots of estimated population structure from *K* = 2 to *K* = 4 (C). Origin and region in Japan of each var. *sinensis* accession is shown to the right of barplots. Dendrogram of Ward’s hierarchical clustering based on Euclidean distance (D). Population structure was analyzed with 12,787 SNPs. Black triangles in (C) and (D) represent ‘Yabukita’.

To understand the ancestral admixture situation of ‘Yabukita’ without the bias of its pedigree, we conducted a Bayesian clustering analysis and HCA of var. *sinensis* excluding the ‘Yabukita’ pedigree (Fig 5 and S1 Table). We inferred the optimal *K* value by plotting *ΔK* (A) and the likelihood at each *K* (Fig 5A and 5B). The Δ*K* values were highest for *K* = 2, followed by *K* = 3 and *K* = 4. At *K* = 3 and *K* = 4, the ancestral barplots showed that ‘Yabukita’ was genetically closer to Chinese accessions than to other Japanese accessions (Fig 5C). This result was also supported by the HCA (Fig 5D). However, the ancestral admixture situation of the ‘Yabukita’ pedigree remained uncertain. We assessed the ancestral admixture situation in the ‘Yabukita’ pedigree based on its ancestral components at *K* = 5 in a structure analysis of Japanese var. *sinensis* (Fig 4A, 4B, and 4C). In most cultivars developed by artificial crossing and natural crossing with ‘Yabukita’ (F1 progeny of ‘Yabukita’), the ‘Yabukita’-type component accounted for more than half (Fig 4A and 4B). In addition, in the two F2 progeny of ‘Yabukita’ (‘Koshun’ and ‘Fushun’) the Yabukita-type component accounted for more than half (Fig 4C).

## Discussion

The ddRAD-seq method is suitable for identifying very large numbers of SNPs in many accessions [16]. This method has been used as an efficient genotyping tool for several different crops [17–20]. In this study, we conducted ddRAD-seq for SNP genotyping of 167 tea accessions with various genetic backgrounds. More than 10,000 SNPs were detected by ddRAD-seq, and were widely mapped across the whole tea genome (Table 1). Based on this high-density SNPs information, we evaluated the genetic structure and the degree of relatedness among tea accessions by the Bayesian clustering approach and multivariate analyses. The tea accessions were classified into the following three subgroups (Fig 1): Japanese var. *sinensis*; Japanese and exotic var. *sinensis*; and Assam hybrids and exotic var. *sinensis*. This result was supported by the results of the ancestral components analyses (Fig 1F).

In a previous study, genotyping of tea accessions by SSR markers revealed marked genetic differentiation between Japanese var. *sinensis* and exotic accessions including var. *assamica* [12]. A possible reason for this result was that there was a bias in the SSR markers that were identified based on polymorphisms between the Japanese and Chinese accessions [12]. Our analyses clarified the genetic structure of each accession without biases from their genetic backgrounds by using high-density SNPs markers.

In our study, most Assam hybrids belonged to ancestry Group 3 (orange color in Fig 1C), suggesting that ancestry Group 3 consisted of cultivars with the Assam-type genome. All the cultivars with ‘Benikaori’ and ‘Iram y5’ ancestry were in Group 3 (S1 Table), revealing that these two accessions were representatives of the Assam type.

The tea accessions analyzed in this study included three Taiwan-yamacha accessions, which are unique tea accessions grown predominantly in the Taiwan highlands. These three Taiwan-yamacha accessions were classified in Group 3, that is, the Assam-type population (S1 Table). Although the genetic background of Taiwan-yamacha is still unclear, its morphological characteristics are more similar to those of var. *assamica* than to those of var. *sinensis* [34]. Previous analyses based on RAPD and AFLP markers indicated that the genetic background of Taiwan-yamacha is dissimilar from both var. *sinensis* and var. *assamica* [11]. To clarify the genetic background of the Taiwan-yamacha accessions, more accessions with a wider range of characteristics and from different parts of Taiwan should be analyzed in further studies.

Ancestry Groups 1 and 2 comprised mainly Japanese var. *sinensis* and Japanese and exotic var. *sinensis*, respectively (Fig 1C and 1F). Many Japanese landraces were in ancestry Group 1, while ‘Yabukita’, and its pedigree, and many Chinese accessions were in ancestry Group 2 (S1 Table). The genetic structure of ‘Yabukita’ differed from those of other Japanese landraces but was similar to those of many Chinese accessions.

There are major phenotypic differences between var. *sinensis* and var. *assamica* [7]. The tea accessions in this study showed wide variations in leaf morphological traits (Fig 2A). There was no significant relationship between genetic structure and leaf morphological traits in the tea accessions, but leaf morphology varied widely within each genetic group (Fig 2B and 2C). These results suggested that differentiation of leaf morphology is not the result of genetic differentiation, and that variation in leaf morphology has expanded within each genetic subgroups of tea plants. Hashimoto (1971) [7] also reported wide variations in leaf area and shape within each variety. In this study, we analyzed Assam hybrids but not var. *assamica*. Further studies on this relationship should include var. *assamica* accessions.

Many tea cultivars and local landraces with different phenotypes are grown in Japan, but little genetic information was available for these lines. We therefore evaluated the genetic structure of Japanese tea landraces and cultivars. Japanese tea accessions were classified into four subgroups mainly corresponding to two subgroups of Shizuoka landraces, Uji landraces, and ‘Yabukita’ and its pedigree (Fig 3). A recent study reported that Uji landraces were genetically separated from landraces from other regions including Shizuoka [13]. These results are in good agreement with the historical background of the introduction of tea plants from China into Japan. The results suggested that Japanese tea landraces in Uji and Shizuoka were introduced from China via different routes. The population analysis of only var. *sinensis* accessions indicated that most Uji and Shizuoka landraces were not grouped with Chinese accessions (Fig 5). It was considered that most of Chinese accessions analyzed in this study were genetically distant to the ancestor of Uji and Shizuoka landraces. To reveal the introduction route from China and origin of Uji and Shizuoka landraces, it is necessary to analyze the genotypes of more tea landraces not only in Uji and Shizuoka but also in China. In this study, we mainly investigated the Uji and Shizuoka landraces, but there are many landraces in other regions of Japan, such as Kyushu, Kinki, and Kochi [9]. To understand the geographical distribution of Japanese tea accessions, it is also necessary to analyze genotypes of many more tea landraces in these regions.

In Japan, ‘Yabukita’ is a leading cultivar for the production of green tea. It is grown in approximately 75% of tea fields and is frequently used as breeding material [3]. Therefore, it is important to understand the ancestral origin of ‘Yabukita’ for tea breeding in Japan. The results of our genetic structure analysis and the HCA showed that ‘Yabukita’ is genetically closer to Chinese accessions than to Japanese accessions (Fig 5C and 5D). In other studies, RFLP analyses of *PAL*, the gene encoding phenylalanine ammonia-lyase, a key gene in catechins biosynthesis, revealed that ‘Yabukita’ has a rare genotype among Japanese tea cultivars [9,35]. These results suggested that ‘Yabukita’ is not an accession that arose during the differentiation of Japanese landraces, but one that retained a Chinese ancestral structure. ‘Yabukita’ was selected from Shizuoka landraces in the early 20^th^ century [3]. The ancestor of ‘Yabukita’ may be identified by further genotype analyses of a large number of Chinese accessions and Shizuoka landraces.

In Japan, the leading cultivar ‘Yabukita’ has been widely used as a breeding material [3], giving rise to many improved cultivars. To clarify the ancestral admixture situation of the ‘Yabukita’-type in ‘Yabukita’ pedigrees, we analyzed 11 artificially crossed and six naturally crossed F1 progenies and two F2 progenies of ‘Yabukita’ (Fig 4A, 4B and 4C). In most cultivars developed by artificial crossing and natural crossing of ‘Yabukita’, the ‘Yabukita’-type component accounted for more than half (Fig 4), suggesting that the genetic structure of ‘Yabukita’ was retained in its pedigree with its excellent traits. The results showed that, at the genotype level, many cultivars in Japan were biased towards the pedigree of ‘Yabukita’. To maintain sufficient genetic diversity for future tea breeding in Japan, it is important to develop a breeding program independent of ‘Yabukita’.

## Conclusions

We genetically analyzed 167 tea accessions including var. *sinensis* and Assam hybrids using SNPs markers identified in a ddRAD-seq analysis. All 167 accessions were classified into three genetic subgroups. Leaf morphology varied widely within each three genetic subgroups. Within the Japanese var. *sinensis* population, the 96 accessions were further grouped into four subgroups. This reflected the geographical background and breeding history. Analyses of the genetic information revealed the ancestral admixture situation of the pedigree of ‘Yabukita’, which is now the leading cultivar of Japanese green tea, and showed that the genome structure of ‘Yabukita’ is clearly different from those of other Japanese accessions. Information obtained using these SNP markers will be useful for not only the selection of breeding materials, but also for genome wide association studies to identify loci associated with important agronomic traits of tea plants.

## Supporting information

S1 Table List of all tea accessions in this study and ancestral components in structure analysis. (XLSX)

S2 Table List of Japanese var. *sinensis* in this study. (XLSX)
